# The Role of Contact Care by Adult Children in Relieving Depression in Older Adult Individuals

**DOI:** 10.3390/ijerph19137981

**Published:** 2022-06-29

**Authors:** Seo-Youn Hong, Jae-Hyun Kim

**Affiliations:** 1 Department of Sport, Leisure, & Recreation, Soonchunhyang University, Asan 31538, Korea; ghdhsy0617@sch.ac.kr; 2 Pi-Touch Institute, Seoul 04511, Korea

**Keywords:** depression, older adult, adult children, care, KLoSA

## Abstract

The purpose of this study is to investigate how contact care by adult children influences the effect of caring for grandchildren on depression in older adult individuals. Studies have shown that caring for grandchildren either increases or decreases the symptoms of depression in older adult individuals, while other studies have shown no effect. The reason for these inconsistent results is that the key control variable, contact care by adult children, has been omitted from these previous studies. An analysis of panel data consisting of observations from 162 older adult respondents in the Korean Longitudinal Study of Aging over the 2008–2016 period confirms that the positive effect of caring for grandchildren on depression in older adults increased as the number of adult children who visited their older adult parents after entrusting their children to them increased. As more of their adult children visited the older adult individuals, the latter were more likely to feel that caring for their grandchildren was healing rather than stressful.

## 1. Introduction

Depression is a common mental disorder affecting approximately 4% of the world’s population [1]. Moreover, 20% of older adult South Koreans experience depression [2]. There are many methods to reduce depression, and previous studies have shown that caring for grandchildren is one such important method. However, these studies have reported inconsistent results.

The effect of taking care of grandchildren on the psychological health of older adults has been an important issue since the 1990s. Some studies have found that older adults who care for their grandchildren have more severe symptoms of depression and a greater deterioration in physical health than non-caregivers [3,4,5,6,7,8,9]. However, other studies have found that taking care of grandchildren reduces the symptoms of depression in this population [10,11,12,13]. Moreover, several studies have found that taking care of grandchildren has no direct effect on depression in older adults [14,15,16].

The reason previous studies have arrived at such inconsistent conclusions may be that none of them controlled for the key variable, that is, contact care by adult children. When adults ask their parents to take care of their children without visiting them, older adults are likely to consider it as stressful. In contrast, if the adults visit their parents after entrusting their children to them, they may consider that caring for their grandchildren is a pleasure [17]. 

Studies on depression in older adults have consistently suggested the importance of social relationships, such as establishing and maintaining networks and social support, as important variables to prevent or alleviate their depression. In particular, these older adults maintain their ideology and effectively handle crises through positive interactions and relationships with family and friends [18].

For geriatrics in East Asia, including those in Korea, relationships with offspring play a hugely important role as they age. Additionally, it is reported that the relationships between older adults and their adult children play an important role in the successful aging of the older adults. Consequently, regarding taking care of grandchildren, it is important to consider family systems [19], especially the unique family systems of Korea.

In Korea, adult children and their parents form mutually reciprocal relationships through grandparent–grandchild care. In leaving the well-being of their young children to their parents, these adult children feel a sense of psychological security. The grandparents, by positively contributing to their grandchildren’s physical and emotional health, feel they can, therefore, depend on their adult children to care for them as they become older [20,21].

In this context, with regard to grandparents taking care of grandchildren, the role of middlemen between them should be considered as a core variable. Unfortunately, existing research ignores this. Therefore, the purpose of this study is to investigate how contact care by adult children influences the potential for less depression among aging grandparents who care for their grandchildren.

Our study makes important contributions to research in this field. First, unlike previous studies, we considered contact care by adult children as the key variable. We did so because the effects of caring for grandchildren on depression in older adults are conditional on the contact care provided by their adult children. Second, we used the Hausman–Taylor Estimation to control for potential endogeneity problems between an error term and caring for grandchildren. For example, elderly citizens with underlying diseases, such as arthritis, neuralgia, or cataracts, may experience more severe symptoms of depression when they care for their grandchildren than healthy individuals. However, this variable is unobservable and, thus, the ordinary least squares (OLS) estimator is biased because it cannot control for this variable. The Hausman–Taylor Estimation provides a means to resolve the endogeneity problem by adopting the appropriate instrumental variable within the model. Moreover, the Hausman test of over-identification ensures the validity of the instrumental variable.

## 2. The Relationship between Depression in Older Adult Individuals and Contact Care Provided by Adult Children

**Figure 1:**
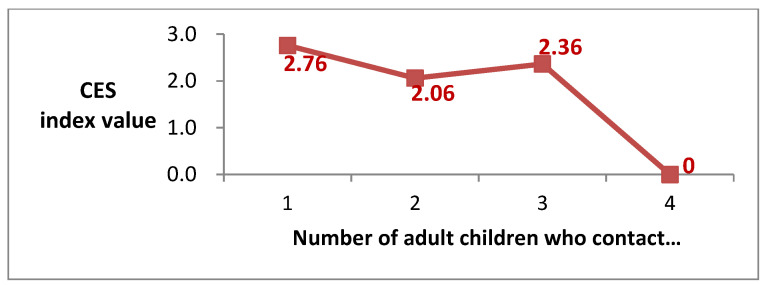
Relationship between CES index values in older adult individuals who care for their grandchildren and contact care provided by adult children. Source: authors’ calculations using Korean Longitudinal Study of Aging data. CES—Center for Epidemiological Studies.

Figure 1 presents the relationship between depression in older adult individuals who care for their grandchildren and the number of adult children who contact their parents more than once a week. We used the Center for Epidemiological Studies (CES) index as an indicator of depression (The CES index, originally published by Radloff [22], is a measure of how often, over the past week, an individual has experienced symptoms associated with depression. The scores range from 0 to 10, with high scores indicating greater depressive symptoms). Based on the average data for South Korea for the period 2008–2016 obtained from the Korean Longitudinal Study of Aging (KLoSA), the CES index values of older adult individuals who care for their grandchildren declined as the number of children who contacted their parents more than once a week increased. This inverse relationship indicates that the positive effect of caring for grandchildren on depression in older adults was stronger when the number of adult children visiting their parents increased. That is, if adult children regularly visited their parents after entrusting their own children to their parents’ care, then the older adult individuals felt that caring for their grandchildren was a pleasure [16,17].

## 3. Empirical Framework

The role of contact care provided by adult children was analyzed as follows:

(1) $$\ln D_{i,t}=c+\alpha_{1}G_{i,t}+\alpha_{2}G_{i,t}\times \ln C_{i,t}+\alpha_{3}\ln C_{i,t}+\beta_{1}\ln S_{i,t}+\beta_{2}\ln O_{i,t}+\beta_{3}E_{i,t}+\mu_{i}+\tau_{t}+\varepsilon_{i,t}$$

where $D_{i,t}$ is the level of depression in older adult individual *i* at time *t*; $G_{i,t}$ is an indicator of whether the older adult individual cares for their grandchildren; $C_{i,t}$ is the number of adult children who contact their parents more than once a week; $S_{i,t}$ is the level of life satisfaction of the older adult individual *i*; $O_{i,t}$ is the level of cognitive ability of the older adult individual *i*; $E_{i,t}$ is an indicator of whether the older adult individual exercises regularly; $\mu_{i}$ is an individual-fixed effect; $\tau_{t}$ is a time-fixed effect; and $\varepsilon_{i,t}$ is a stochastic error term.

Our variables of interest were the indicators of whether an older adult individual cared for their grandchildren, the amount of contact care provided by adult children, and an interaction term for both variables. As mentioned above, inconclusive results have been found in previous studies regarding the effects of caring for grandchildren on the symptoms of depression in older adult individuals, because these studies have failed to consider the key variable: the amount of contact care provided by adult children to their grandparents. Thus, we included the interaction term and the individual terms to show that the effects of caring for grandchildren on depression in older adult individuals are conditional on the amount of contact care provided by their adult children.

We introduced three control variables: the level of an older adult individual’s life satisfaction, the level of an older adult individual’s cognitive ability, and an indicator of an older adult individual’s regular exercise. These variables were chosen based on data from previous studies, as discussed in the following paragraphs.

Lue, Chen, and Wu [23] showed that older adult women with greater life dissatisfaction are more likely to experience depression. Additionally, life satisfaction in men is strongly associated with an absence of anxiety and depression [24]. Altun and Yazici [25] confirmed that the degree of life satisfaction is a significant predictor of depression.

Older people with low cognitive function are more vulnerable to depression than those with high cognitive function [26,27,28]. Reduced cognitive function is significantly associated with depression [29,30,31].

Several studies have shown that regular exercise reduces depression by increasing the release of neurotransmitters, such as dopamine and serotonin, in the brain [32,33,34]. Lindwall, Rennemark, Halling, Berglund, and Hassmén [35] confirmed that older adults who do not exercise have higher depression scores than those who perform either light or vigorous exercise. Tsang et al., [36] found that a 16-week qigong exercise program decreased the symptoms of depression in 82 senior citizens.

## 4. Empirical Methods and Data

Various econometric methods can be used to estimate Equation (1), such as the OLS, fixed-effect, random-effect, and Hausman–Taylor methods. However, OLS estimation may be biased, because it does not control for each individual’s specific characteristics, and although fixed-effect and random-effect analyses provide suitable alternatives to control for these individual characteristics, they may be not suitable in our case because the explanatory variables may be correlated with an error term (these endogenous problems are described in the following paragraph). Thus, we used the Hausman–Taylor method.

First, omitted-variable bias can occur. For example, compared to healthy older adult individuals, older adult individuals with underlying diseases, such as arthritis, neuralgia, or cataracts, may experience more severe symptoms of depression when they care for their grandchildren. However, the variables related to these health conditions are inaccessible because they are non-disclosed information. Second, simultaneous bias can occur. Baune, Miller, McAfoose, Johnson, Quirk, and Mitchell [37] showed that people with depression have poorer cognitive abilities in all domains than healthy people. This reverse causality may cause bias.

The Hausman–Taylor estimation provides a means to resolve the endogeneity problems by using an appropriate instrumental variable in the model. Moreover, the Hausman test of over-identification ensures the validity of the instrumental variable. Thus, the null hypothesis of the Hausman test (based on a comparison of the fixed effects and Hausman–Taylor estimations) should not be rejected.

We used survey data from South Korea for the period 2008–2016, collected by the Korea Employment Information Service, to estimate Equation (1). The CES index values were obtained from answers to the 10 questions listed in Table 1. Each respondent gave a score of 1 for “yes” or 0 for “no” when answering each question. The final CES index score was obtained by summing the scores for each question. Thus, a higher score on the CES index indicates a higher level of depressive symptoms.

Table 2 presents the survey questions for the explanatory variables. An indication of whether the individual cared for their grandchildren was obtained from the question, “Do you take care of grandchildren aged younger than 10”? The score for contact care provided by adult children was obtained from the question, “How many adult children visit you in a week”? The level of life satisfaction was obtained from the question, “Compared to your peers, how satisfied are you with your overall life”? Cognitive ability was determined using the question, “What is your level of cognitive ability for memory, numeracy, and language skills”? Regular exercise was assessed using the question, “Do you exercise more than once a week”? Table 3 reports the summary statistics of the variables.

## 5. Empirical Results

Table 4 shows the regression results of the specification comprising the control variables obtained from previous studies. The coefficients, lnS and lnO, are negatively significant at the 1–10% level for all models. These results indicate the validity of the results from previous studies. The value of coefficient E in the OLS model is negatively significant at the 10% level. However, the values of the coefficients in the fixed-effect and Hausman–Taylor models are positive but insignificant. The value of the E variable coefficient from the OLS analysis is consistent with the values of the coefficients that have been reported in previous studies. The null hypothesis for the over-identification test of the Hausman–Taylor analysis is not rejected. Thus, the instrument variables for the Hausman–Taylor estimations are valid.

Table 5 shows the regression results of Equation (1). The value of the coefficient of G was positively significant at the 5% level for the fixed-effect and Hausman–Taylor models. The value of the term representing the interaction between G and lnC is negatively significant at the 1% level in all models. The coefficient of lnC has an insignificant positive value in all models. Therefore, the signs of the coefficients for the variables of interest are consistent with our expectations.

The results of the control variables are consistent with the results presented in Table 4. The null hypothesis for the over-identification test of the Hausman–Taylor analysis is not rejected. Thus, the instrument variables for the Hausman–Taylor estimations are valid.

**Figure 2:**
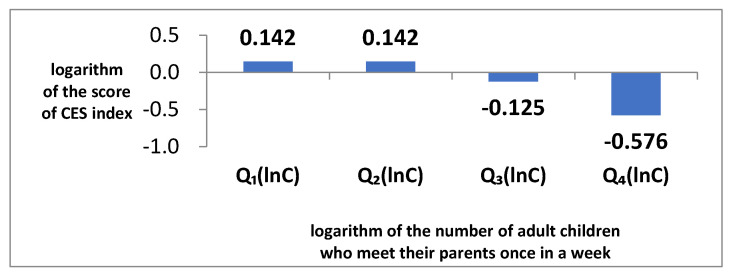
The effect of contact care by adult children at relieving depression in older adult individuals based on the lnC value. Note: $Q_{i}\left(\mathrm{lnC}\right)$ indicates the first quartile of lnC. The *x*-axis is the logarithm of the number of adult children who visit their parents once a week. The *y*-axis is the logarithm of the Center for Epidemiological Studies (CES) index score. Source: authors’ calculation using the result of empirical analysis.

Figure 2 shows the effect of contact care by adult children on relieving depression in older adult individuals based on the lnC value. Based on the result of the Hausman–Taylor model, the effects of G are 0.142, 0.142, −0.125, and −0.576 for the first (0.69), second (0.69), third (1.10), and fourth quartiles (1.79) of lnC values. This suggests that the effect of caring for grandchildren on depression in older adult individuals increases the more adult children visit the older adult individuals.

Table 6 shows the results of the robustness tests of the regression results reported in Table 5. We confirmed the main results by re-estimating Equation (1) after changing the indicator values. We used the number of adult children who visited their parents more than once a week as the contact care value for the baseline model. When the number of adult children living with their parents increases, the older adult individuals should feel that caring for their grandchildren is a pleasure. Therefore, we replaced “the number of adult children who visit their parents once a week” with “the number of adult children living with their parents”.

We used a dummy variable as the variable for older adult individuals caring for their grandchildren in the baseline model. To confirm that the same result was obtained even if the dummy variable was replaced with a numeric variable, we replaced the “indicator of whether the older adult individual cared for their grandchildren” with “the number of children who are cared for by their grandparents”.

The results of the robustness test show that the value of the coefficient of the term representing the interaction between lnG and lnC is significantly negative at the 10% level for the fixed-effect and Hausman–Taylor models. In contrast, the value of the coefficient of lnG is significantly positive at the 5–10% level in all models, and the value of the coefficient of lnC is significantly positive at the 1% level in all models. Note that the value of the coefficient of lnC is significant in this model, but not in the baseline model. The values of the coefficients of lnS and lnO are insignificant, but the signs of the coefficients are the same as those in the baseline model. The value of the coefficient of E is insignificant, as it is in the baseline model. These results for the variables of interests are consistent with the results of the baseline model. In summary, it can be concluded that the main regression results of this study are robust.

## 6. Conclusions

This study makes an important contribution to the literature on the treatment of depression in older adults. Some previous studies have shown that caring for grandchildren either increases or decreases symptoms of depression in elderly grandparents, while others have shown that doing so has no effect. The reason for these inconsistent results is that a key control variable, contact care by adult children, has been omitted from these studies. Therefore, in this study, we determined whether depression is lessened in this population based on whether contact care is provided by adult children to their parents (i.e., the geriatric grandparents). Panel data consisting of observations from 162 older adult respondents in the KLoSA over the 2008–2016 period confirmed that the effect of caring for grandchildren on depression in older adult individuals was 0.142, 0.142, −0.125, and −0.576 for the first (0.69), second (0.69), third (1.10), and fourth (1.79) quartiles of the logarithm of the number of adult children who visited their parents once a week. This suggests that the treatment effect of caring for grandchildren on depression increases as the number of adult children who visit their parents increases. That is, if more adult children visit their parents, the latter feel that caring for their grandchildren is healing rather than stressful.

In general, an increase in exchanges and communication between generations is closely related with a decrease in symptom of depression and suicide [38,39]. Considering the collectivistic culture and emphasis of interdependence between generations in Korea [40], active interactions of grandparents with their adult children were important rewards leading to positive mental health, as found in other family societies such as Taiwanese and Chilean [11,15]. Their depression does not decrease solely by having regular contact their with grandchildren, but by frequent, active exchanges with adult children facilitated by said childcare. This is the core variable that leads to a significant decrease in their depression. This research implies that to understand the essence of taking care of grandchildren, it is necessary to consider the family system, including the relationship with adult children as well, as the relationship with grandchildren.

Medication, psychotherapy, and family therapy are generally used in the psychiatric treatment of older adults. This study shows that caring for grandchildren is effective as a method of family therapy for the treatment of depression in the elderly, provided that the latter are also visited by their adult children. Therefore, psychiatric hospitals should use caring for grandchildren as a form of family therapy, and they should encourage adult children to visit their parents. When developing local governmental policies and providing services for grandparents, it is necessary to emphasize relationships within the entire family unit. Evidently, taking care of grandchildren increases contact with adult children, and policies and education focused on this type of childcare should emphasize such aspects.

Our study has a limitation that should be noted. First, the number of daily hours grandparents spent with their grandchildren and whether they lived with their grandchildren could not be determined, and was not included in our analyses. Additionally, we used an indicator of whether older adult individuals cared for their grandchildren in the baseline analysis and the number of grandchildren who were cared for by their grandparents in the robustness analysis. However, these indicators do not contain information about the effort level required for the grandchildren’s childcare. This information may influence the effect of caring for grandchildren on depression. We will aim to solve this problem in future studies once we obtain these data.

## Figures and Tables

**Table 1:** Survey questions used to measure the Center for Epidemiological Studies (CES) index.

Component	Survey Questions
CES Index	Sum of the scores for 10 questions about depression
1	Did people seem cold to you during the past week?
2	Were you sad during the past week?
3	Did you feel depressed a lot during the past week?
4	Did everything seem hard during the past week?
5	Do you think you were not relatively well during the past week?
6	Did people seem to hate you during the past week?
7	Do you think you didn’t sleep well during the past week?
8	Did you have major problems performing tasks or activities during the past week?
9	Did you feel alone in the world during the past week?
10	Were you unable to do anything during the past week?

Source: Korea Employment Information Service data.

**Table 2:** Survey questions to assess explanatory variables.

Component	Survey Questions
Indicator of whether the individual cares for their grandchildren	Do you take care of grandchildren aged younger than 10?
Score for contact care by adult children	How many adult children visit you in a week?
Level of life satisfaction	Compared to your peers, how satisfied are you with your overall life?
Level of cognitive ability	What is your level of cognitive ability for memory, numeracy, and language skills?
Indicator of regular exercise	Do you exercise more than once a week?

Source: Korea Employment Information Service data.

**Table 3:** Summary statistics.

Classification	Variable	Observation	Mean	Standard Deviation	Minimum	Maximum
Dependent	lnD	775	1.21	0.78	0	2.40
Independent	G	775	0.13	0.34	0	1
	G × lnC	775	0.12	0.33	0	1.61
	lnC	775	0.94	0.29	0.69	1.79
	lnS	775	4.11	0.35	2.30	4.61
	lnO	775	3.20	0.30	1.10	3.43
	E	775	0.39	0.49	0	1

Source: Authors’ calculations using Korea Employment Information Service data.

**Table 4:** Regression results for Equation (1) without the variables of interest.

DV: lnD	OLS	FE	HT
lnS	−0.758 ***	−0.471 ***	−0.471 ***
	(−10.29)	(−5.54)	(−5.54)
lnO	−0.661 ***	−0.221 *	−0.221 *
	(−7.74)	(−1.75)	(−1.75)
E	−0.089 *	0.043	0.043
	(−1.70)	(0.70)	(0.70)
No. of observations	775	775	775
*R* ^2^	0.264	0.393 (within)	-
$\chi^{2}$ (168)	-	-	1033.6 ***
$\mathrm{Hausman}\;\mathrm{over}-\mathrm{identification}\;\mathrm{test}:\;\chi^{2}$ (7)	-	-	0.00

Note: *, and *** represent significance at the 10%, and 1% levels, respectively. Values in brackets indicate a *t*-value or *z*-value. The estimated values for the coefficients for year-fixed effects and constants are not reported in the tables, but they were included in the analysis.

**Table 5:** Regression results for Equation (1).

DV: lnCES	OLS	FE	HT
G	0.334	0.593 **	0.593 **
	(1.38)	(2.47)	(2.47)
G×lnC	−0.562 **	−0.653 ***	−0.653 ***
	(−2.25)	(−2.75)	(−2.75)
lnC	0.096	0.778	0.778
	(1.03)	(0.74)	(0.74)
lnS	−0.767 ***	−0.446 ***	−0.446 ***
	(−10.44)	(−5.51)	(−5.51)
lnO	−0.621 ***	−0.220 *	−0.220 *
	(−7.19)	(−1.75)	(−1.75)
E	−0.093 *	0.050	0.050
	(−1.79)	(0.82)	(0.82)
No. of observations	775	775	775
*R* ^2^	0.276	0.389 (within)	-
$\chi^{2}$(171)	-	-	1049.2 ***
$\mathrm{Hausman}\;\mathrm{over}-\mathrm{identification}\;\mathrm{test}:\;\chi^{2}$(10)	-	-	0.00

Note: *, **, and *** represent significance at the 10%, 5%, and 1% levels, respectively. Values in brackets indicate a *t*-value or *z*-value. The estimated values for the coefficients for year-fixed effects and constants are not reported in the table, but they were included in the analysis.

**Table 6:** Regression results for the robustness tests.

DV: lnCES	OLS	FE	HT
lnG	2.856 **	2.28 *	2.28 *
	(2.04)	(1.90)	(1.90)
lnG × lnC	−3.175	−3.074 *	−3.074 *
	(−1.66)	(−1.86)	(−1.86)
lnC	4.768 **	5.714 ***	5.714 ***
	(2.35)	(3.16)	(3.16)
lnS	−1.075 **	−0.177	−0.177
	(−2.58)	(−0.37)	(−0.37)
lnO	−3.027 ***	−0.991	−0.991
	(−5.14)	(−0.98)	(−0.98)
E	−0.180	−0.127	−0.127
	(−1.01)	(−0.64)	(−0.64)
No. of observations	50	50	50
*R*^2^ value	0.634	0.128 (within)	-
$\chi^{2}$ (171)	-	-	138.6 ***
$\mathrm{Hausman}\;\mathrm{over}-\mathrm{identification}\;\mathrm{test}:\;\chi^{2}$ (10)	-	-	0.00

Note: *, **, and *** represent significance at the 10%, 5%, and 1% levels, respectively. Values in brackets indicate a *t*-value or *z*-value. The estimated values for the coefficients and constants are not reported in the table, but they were included in the analysis.

## Data Availability

All relevant data are within the manuscript.
